# Mercurial Necrosis Resulting from Amalgam Fillings

**Published:** 1899-07

**Authors:** J. Y. Tuthill

**Affiliations:** Brooklyn, N. Y.


THE
AMERICAN JOURNAL
—OF—
DENTAL SCIENCE.

VOL. XXXIII. BALTIMORE, JULY, 1899. NO. 3.

### Selections.

ARTICLE I.

### MERCURIAL NECROSIS RESULTING FROM AMALGAM FILLINGS.

BY J. Y. TUTHILL, M. D., BROOKLYN, N. Y.

**Read before the Medical Society of the County of Kings.**

Mercurial necrosis is a fiield of pathology which has not received the investigation it deserves by the medical profession. While the poisonous effects of mercury have so long been recognized that I need take no time in rehearsing them, yet there are certain causes affecting the nerve centers which demand more thorough investigation than has yet been given.

In presenting this subject to the Society for consideration I want to show that by the use of amalgam in filling teeth there is a possibility of mercurial poisoning, which seriously affects the nerve-centers, impairs locomotion by heaviness of limb and stiffness of joint, gives rise to obstinate diseases of the skin, and makes a mental wreck of its victim, whose imaginations and hallucinations are more than my pen can describe.

Physical examination reveals nothing to assist the physician in making a diagnosis of his case, as all the functions are usually well performed. There is, however, nervous depression, irritability, unreasonableness, an inability to overcome and throw off feelings of oppression which settle upon the patient and hold him as in the clutches of despair until his ambition is broken, his energy is gone, his purpose is lost, and he drifts for lack of power to concentrate his actions and assert himself as a force in the world. There are shifting, shooting pains here and there from head to foot, affecting sometimes one part and then another; numbness of hand, foot, or jaw, heaviness of leg, arm, or head, with an almost inability to move them, and a feeling as though one would fall or lose consciousness. Again there is a light, floating sensation as though moving in air. There is mental excitiability as well as mental depression; perplexing events cause the highest degree of excitement, ordinary conversation sometimes causes complete confusion, headache, palpitation, intense solicitude, and anxiety, without reason for it. Such are some of the symptoms attending these cases.

To bring this pathological condition more clearly to your thought, allow me to cite a few cases which have come under my observation during the past few years, and you will pardon me for alluding to my own individual case which first opened my eyes to this subject.

During the winter of 1884 and 1885, when working far beyond the limits of good judgment, and averaging from October 15th to April 27th, not more than from four to four and one half hour's sleep per night, I was attacked February 1st with neuralgic and rheumatic pains, usually short in duration, lasting from ten to fifteen minutes to three or four hours, affecting chest, back, arms and legs. My work was arduous, and the professional demands upon my time and strength prevented my getting adequate rest.

With failing strength and energy, I continued my daily routine of work until April 27th, when I was in a state of physical and mental collapse. I sought a quiet place for rest, and April 28th went to Saratoga Springs. Arrived there at 3 p. m., and in a half an hour was in bed. I had a good night's rest, and after breakfast the following morning took a stroll through the village, which at this season of the year is the sleepiest place of its size that I know of. Then, thought I would write home news of my safe arrival and a good night's rest. But never was I more surprised than to find that I could not write more than two or three words when my hand would be so numb that I could not hold the pen, until I had rubbed it for some minutes. The same processes were repeated over and over again until I succeeded in writing a short letter. This condition continued, with some abatement, for many weeks and, in much less degree, for years following. I remained in Saratoga for ten days, sleeping thirteen and fourteen hours in every twenty-four, then returned home and resumed practice feeling much improved.

An eruption which appeared like psoriasis, and which had troubled me for several years, became more troublesome and refused to heal, much to my annoyance and chagrin. All the foregonig conditions continued up to the spring of 1889, when a persistent headache, often of neuralgic character, continued for two or three months was relieved by taking a four months' trip abroad.

Since that time I have had average health, with occasional numbness of hands. The handling of steel would almost paralyze my hands for some minutes to come. The eruption continued the same, resisting all treatment that my own ingenuity could devise or that my professional brethren, who took a kindly interest in my case, suggested

The numbness attended with pain in the fingers, was thought to be a form of gouty neuritis. Following their advice, I took such remedies as they prescribed, sometimes it seemed for the better, but with no permanent improvement.

In the summer of 1895, spending the month of August on the Shawangunk Mountains, I was applying to the eruption several times a day, a lotion of bichlorid of mercury, which accomplished more for healing than anything I had ever used; but while the eruption was fast getting better, my hands were rapidly losing their power and I could not rely on holding anything, nor could I pick up small things like pins, needles, or twine, because of the numbness. At this juncture, it occurred to me that I was absorbing mercury, and that mine was a case of mercurial neuritis or paralysis, the result of this absorption, and that the eruption was a mercurial psoriasis. Returning to the city I laid the matter before several physicians, who thought I was mistaken, as my gums showed no evidence of mercurial poisoning, and on their advice resumed the use of the bichlorid loition, but was compelled to abandon it in a few days because of numbness which followed. I reported my condition. They then agreed with me that the mercury affected my system in an unusual manner, but thought that I had an idiosyncrasy, and that it would probably not affect more than one in a thousand under the same circumstances.

Being thoroughly aroused as to my state, and having several amalgam fillings in my teeth, I soon came to believe that my entire condition, the numbness, the rheumatic pains, and the eruptions were all due to the action of mercury absorbed from those fillings. So I decided to have them removed, and the work was done in May, 1896. In two weeks I felt like another man; it seemed as though a great weight had been lifted, and I was once more free from an oppression which had hung over me like one long nightmare for years, handicapping, restricting, and restraining me on every hand.

My improvement has continued from that time to the present, the numbness growing less and less, the eruption disappearing until there is less of it than at any time in fifteen years.

I do not magine that all the mercury that I absorbed from those fillings which I carried or thirty-eight years; is out of my system, but from my general improvement, believe it is growing less all the time, and I am feeling better than at any time since the spring of 1885. Having lived for eleven years on the ragged edge of hope and despair, and thus secured by freedom from a bondage worse than human slavery, I could appreciate the sufferings of others when they rehearsed experiences which I had realized. As a natural consequence, I readily recognized the same enemy to their peace and happiness which had shattered my own.

In December, 1896, I was called to see Miss F., aged thirty-three years, who had been in excellent health previous to an attack of la grippe in December, 1892. Since then her nervous system had been much disturbed, and she had come to be melancholic and to withdraw herself from her family and friends, seeking the seclusion of her room—refusing to go out or to associate with others, or even with the members of her own household. When I was called, she had been treated by five different physicians with no improvement. I treated her for indigestion, torpid liver, constipation, etc., for ten weeks, with no improvement in her mental condition. Then numbness of hands and stiffness of jaws led me to examine her teeth, when I found five amalgam fillings which I believed had produced a mercurial neurosis. I gave it as my opinion that her condition was due to the effect of mercury upon her nervous system absorbed from those fillings and advised their removal. She was stubborn and for some time refused, but yielded in May, 1897, and had the work done. She has steadily improved since that time, and her family say that she has not been so well in five years as now.

Case 2. In the early part of last September, Miss E., aged eighteen, was brought home from a three-months sojourn in the country, with all the indications of typhoid fever. Intestinal antiseptics, tonics, etc., arrested the progress of the case, but an unaccountable languor, debility, loss and flabbiness of flesh with drowsiness, continued for several weeks, she sleeping eighteen and twenty hours out of twenty-four. My case otherwise appearing well, her sluggish gait, heaviness of limbs, and stiffness of jaws, led me to examine her teeth, in which I found nine amalgam fillings. Being convinced that these were retarding her recovery, I advised their removal and the substitution of gold. When the fillings were taken out she became more wakeful and animated, and has continued to improve although still suffering from the effects of the absorbed mercury.

Case 3. Miss K., a young lady of culture and refinement, was brought to my office December 1st, 1887, suffering from extreme nervousness, which had continued for three years. She was restless and could not apply herself for any length of time to any one thing, sleepless, irritable, hysterical, etc. Having made a thorough examination of her case and being assured that all her functions were normal, I examined her teeth and found sixteen amalgam fillings, several of them in teeth containing gold fillings. Believing this to be a case of mercurial neurosis I told her, and her mother who accompanied her, that the case put me in mind of what I had read in "Ziemssen's Cyclopædia" a day or two previous on mercurial poisoning, and I read to them, viz.: "Essentially the condition is characterized by great mental excitability of the patient to external impressions. Every unexpected or perplexing event excites him in the highest degree. The visit and the conversation of the physician put him into a state of complete bewilderment, even to syncope; the adult patient grows pale and stammers in answering the simplest questions. To perform his allotted task requires the greatest effort, or is even impossible if he sees or thinks he is being watched. There is also great solicitude and a feeling of anxiety without any reason for it. There is sleeplessness, or sleep which is restless, frequently broken and disturbed by frightful dreams, headache and papitation. In the severer forms there are frequently hallucinations, usually of a frightful nature. When perplexed or excited, traces of tremor are often perceptible in a slight twitching of the muscles of the face at the corners of the mouth." Having heard this, she remarked that it was a perfect description of her case in every particular, except that she had not had the hallucinations there mentioned. Following my advice the fillings were removed and the young lady has improved very rapidly to the present time, all her nervous feelings having disappeared. Indeed, her mother tells me that she manifested none of her nervous troubles since the removal of the fillings.

Case 4. In February, 1897, was called to see Mrs. N., who was extremely nervous, with neuralgic pains in the chest and palpitation of the heart. Upon looking up she felt as though she were falling backward. Had heaviness of limbs, loss of memory, and found it difficult to think and harder still to put her thoughts into words. Numbness of hand or foot, a sensation of floating, and a feeling that she would die, were common experiences. I did not see her many times before I examined her teeth, and found one large amalgam filling in a lower molar, which I advised her to have removed, but doubting its necessity she kept it until November. Here I wish to read a letter sent me several weeks ago:

DEAR DR. TUTHILL:

"I wonder if there is another woman in this world who has had all the peculiar feeling that I have had within the past two years? You know that I am one of the healthiest-looking mortals, have good appetite, sleep fairly well, etc., yet for all this time I have suffered from such awful feelings. I was afraid to go out alone, or be alone, and in constant fear that something dreadful was about to happen. I suffered much from palpitation. Sometimes, when sitting in a street-car, the people would suddenly begin to look queer, the car crooked, and I would look around at each one to see if any one realized the dreadful condition of things. I had also light, floating sensations, and at times it was hard to talk, to think, and speak in sentences. When lifting my eyes upward I felt as though I were falling backward. Then my limbs were so heavy, my memory seemed to be gone, and I often felt dazed. Even when conversing with friends, or sitting at the dining-table, such a horrible feeling would suddenly seize me and I thought that in a moment I might fall down dead. I became hot and cold and sick, and would have to rise and walk about till it passed over. The feeling that I was just about to die often came to me with sensations that I cannot describe, but such as I should imagine a person dying would have. It began at my feet, a numb, cold creeping feeling, and seemed to be hardening all over. Two or three of these spells I would have in a day, lasting from ten minutes to half an hour.

"You told me last spring that if I would have an amalgam filling in one of my teeth removed, a large part and perhaps all of these nervous troubles would disappear. But it seemed to absurd to me that I hardly gave it a thought till you urged it again this fall. While my husband was in one of the Western cities he happened to be in a large Dental Association and asked the president if he had ever heard of amalgam fillings causing nervous troubles. He replied, 'Yes we have. It is not common, but some people are poisoned by the mercury, as I can prove,' and then cited the case of a man in that city who was a nervous wreck, given up by several physicians. At last one doctor said he believed him to be suffering from mercurial poisoning, and, upon examination, found seven amalgam fillings in his teeth. They were removed and from that day the man began to improve, and is a strong, well man to-day, scarcely knowing what a nerve is.

"So my husband wrote to me to take your advice and have the filling removed. I did so about two months ago. Immediately I felt better and coming home felt as though

I could have walked to Beersheba and not have fallen. My buoyant feelings have not lasted, however, and some days I am almost discouraged. But I know that I am better. I have not had one of those awful dying sensations, nor do I have nearly so much of my nervous troubles. I can go downtown and to New York and do many things that I could not do before I had the amalgam filling out. Still I do have days of some of the old feelings and fears, but they pass away more quickly. I am living in hopes that it will be as you said, 'They will all be gone to stay away, in time.' But as the filling was in two and a half years, I could not expect to be cured in two months.

"Oh, Doctor, how I wish I had taken your advice last spring and saved myself the sufferings of the summer and fall! I wish I had never had it put in my mouth. No dentist would put in another for any amount of money. I want to thank you with all my heart for insisting upon my having that filling removed and bringing brightness again into my life. Days when I feel well I am the happiest woman living. I only long to feel entirely well, and trust as the poison passes off that I shall.

"Very gratefully yours,
Mrs. N."

Case 5. In July last I was called to see Mrs. H , aged twenty-six, who was in a very excitable condition, afraid to go out alone in the street or stores lest she become unconscious and be taken to some hospital. Upon looking up she felt as though she were falling backward; when looking down, as though falling forward; when standing still, as though she were going to pieces; when lying in bed, as though floating toward the side wall; when sitting in a chair she often felt as though she were dead all over, and that it would require the greatest effort to make any movement. Her troubles began in the fall of 1895, with pains in the back of her neck and about the lumbar regions followed by pains in her left thigh and arm, which would last from a minute to one or two hours and then pass off to reappear there or elsewhere at irregular intervals. In the spring of 1896 she complained of stiffness of the jaws, of her left hand and foot going to sleep, with continued numbness of the third finger of the left had. The left side of her head and the left ear would often become numb. The large toe on the right foot would be numb for weeks at a time. She also complained of her limbs being heavy, like lead, and at times it was difficult for her to raise them. There was loss of memory, the eyes looked dull and heavy, the skin had a dingy look which washing did not improve. Feelings of dread and fear lest some calamity might befall her, made her afraid to stay in or go out alone, which she had ceased to do for more than a year. I treated this lady for several weeks without making any progress on the case. Then I examined her teeth and found nine amalgam fillings, which I believed had more to do with her condition than anything else. I therefore advised the removal of the fillings and that they be replaced with gold or bone. The work was done. In less than a week she began to improve; in three weeks you would hardly have believed her to be the same woman. All her symptoms have abated; the numbness, the heaviness of limbs, the constant fear, the falling tendencies, the stiffness of jaws, have disappeared, and she goes out alone. There is a buoyancy and vivacity in her manner which shows that her hopes and anticipations are bright. Her skin looks clear and healthy, her eyes sparkle with expression, while her memory is true to her as in former days. Since the removal of the amalgam she has gained twenty pounds in weight. I might say in passing that nine physicians had treated this case before it came to me.

I might describe several other cases, in one of which the fillings were in between twenty-five and thirty years, and the toxic effects manifest for a dozen years; but those already given are a sufficient illustration.

Members of the dental profession who are so freely using amalgam tell us that over the surface of each filling there is an oxidation which prevents any possible absorption of the mercury. Fillings which have been removed are bright, where in contact with the cavity, indicating that this oxidation occurs only to the exposed surface and not to that which comes in contact with the structural parts of the teeth. The circulation in the teeth is continually in contact with the unoxidized surfaces of the amalgam and is constantly receiving some mercurial taint which is carried throughout the system. As the nerve-centers are most impressionable to its toxic effects, we find neurasthenic conditions chiefly resulting.

It is not my purpose at this time to indulge in theories as to how these results follow the use of amalgam, but to merely state the facts. While many having these fillings seem to be exempt, others suffer from the subtle effects of the mercury. This is clearly proven by the cases I have cited, which have come to my knowledge during the last two years. So long as the system keeps in vigorous condition many feel no ill effects of the poison, but when from any cause it falls below par, either from over-tax or from disease, the toxic effect of the mercury becomes dominant, with those susceptible to it, and gets the mastery of the nervous system, to be followed in many cases by the train of symptoms mentioned above.

Although the number of cases may be comparatively few, they deserve as thoughtful consideration as would be given the subject if mercurial neurosis were more common. I doubt not that our insane asylums have many an inmate of a mental state developed by amalgam fillings, which produces excitation or sluggishness of brain, impairs thought, destroys memory, blunts perception, and relegates to despair what otherwise might be a bright and brilliant career.

To insist upon a patient's enduring the agony, torture, and expense of having these fillings changed for gold, requires considerable nerve, with positive assurance of a correct diagnosis, for in case no improvement should follow the physician would be a subject for wholesale condemnation, and branded as a crank. Fortunately for the writer, in no instance has he dvised the removal of these fillings where a marked mprovement has not followed in a few days. It would be unreasonable to expect the relief to be complete and immediate, for the removal of the fillings does not remove the mercury which has been gradually absorbed into the system during months and years of contact with the amalgam, and which has seriously affected the nerve-centers. But it does stop the supply, and when that is cut off there is an abatement of all the more prominent symptoms, followed by a marvelous improvement, and the patient realizes that he is living under new conditions, with hopes and aspirations he has not known for long months and perhaps for years past.

For the conscientious physician to obscure and unsuspected causes of disease have a peculiar interest, and he will not allow prejudice to prevent a careful investigation of the facts which may reveal such causes.

If this paper shall but stimulate some present to a more earnest study of this subject it will have fulfilled its mission and put into motion a train of thought which must eventually secure to many a now hopeless sufferer a relief so great as to be almost the beginning of a new life.

DISCUSSION.

When our President asked me to take part in this discussion, and told me that I would be allowed ten minutes, I determined I could cover more of the enormous ground involved, and in a more connected manner, if I should read what I had to say.

About the year 1826, M. Traveau, of Paris, France. advocated the use of what he called "Silver Paste" for permanent fillings in teeth. This metallic preparation was first brought to the notice of the dental profession in the United States about seventy years ago through the advertisements of two Frenchmen by the name of Crawcour.

It was called by them the "Royal Mineral Succedaneum;" succedaneum, a replacer or substitute, a name which is indicative of fraud and which consequently stamps the adventurers as unworthy of professional respect.

It was soon proven that instead of being a mineral compound it was purely metallic, and consisted of silver and copper rendered temporarily plastic by the addition of mercury. It was easily manipulated, and they were enabled to fill a class of largely decayed teeth with frail and broken cavity walls such as had never been attempted by the most skilful surgeons.

Scientific investigation at last was imperative, and, as a result, the foremost men who had been arrayed against it now became its ardent supporters. Professor Elisha Townsend, one of the best gold-workers of his day, and President of the American Society of Dental Surgeons, found that the cry of mercurial ptyalism was not supported by fact.

This investigation gave birth to an organization called "The New Departure Corps," and was composed by the following gentlemen: Professors Henry Morton and M. B. Snyder, scientists; Messrs. Jacob B. Eckfeldt and Patterson Dubois, assayers of the Philadelphia Mint, metallurgists; Drs. S. B. Palmer, Henry S. Chasee and J. Foster Flagg, dentists. The work of this organization gave to our profession what is known as the new-departure creed, the most important deduction of their work being expressed in the following statement: "In proportion as teeth need saving, gold is the worst material to use."

These gentlemen did much to improve the formula for amalgam alloys and it has gone through successive changes until now we recognize the following to be as good in all respects as we can get: Silver from 60 to 70; tin, from 30 to 35; and gold and zinc from 5 to 10. These are about the proportions. These metals are melted in a crucible and thoroughly stirred while in a molten condition, and then poured into a mold. After the mass has become cold it is cut into fillings with a file and we then have the alloy.

To make an amalgam for filling a cavity the required amount of alloy is placed in one side of a pair of weighing scales, and an amount of mercury in the other sufficient to balance the scales. Both the alloy and mercury are now put into a mortor and very thoroughly mixed, after which all possible mercury is squeezed out through chamois with heavy pliers, and the amalgam is now ready for insertion into a *properly prepared cavity.*

An ordinary sized cavity will require 5 grains of alloy. After adding 5 grains of mercury and thoroughly mixing, 1½ grains of mercury can be expressed through the chamois, leaving 3½ grains of mercury in a filling of 8½ grains. This is in a cavity with entire walls. In cavities where contour fillings are required the amount of mercury will be slightly increased.

The object of putting an excess of mercury in the mixing, and then expressing the surplus, is to facilitate a complete and thorough amalgamation, while expressing of the surplus removes only the mercury, no portion of the other metals passing through the chamois.

Such a filling placed in a *properly prepared cavity* makes what all dentists recognize as a good filling, and from which no mercury can be removed so long as it remains in the mouth. This, I believe to be the consensus of opinion of all scientific practitioners of dentistry throughout the world.

It is claimed, however, that the mercury *does* get out and is absorbed into the system, producing mercurial neurosis, ptyalism, etc.

If any of the mercury could be removed from such a filling it would no longer be a fit stopping for a tooth. It would be disintegrated, soft, and would fall out, either by mastication or the toothbrush. We do not however, find amalgam fillings acted upon in this way. Fillings that have done good service for thirty years are always as hard as the day they were inserted. The mercury *can* be removed by heat and by chemical affinity, neither of which can be produced so long as they remain in the mouth. The boiling-point will no eliminate it, and as the tissues of the mouth will not tolerate anything approaching that temperature, heat is excluded.

I am reminded here of a letter recently received from Dr. Bogue, in which he says, in reply to my question about the action of iodide on amalgam: "I did not make any experiments at all relative to the systemic effects of iodine upon mercury which had entered the system from amalgam fillings in the teeth—perhaps because most of the patients for whom I had the honor of operating have not during my time been subjected to a dull red heat, which is about the temperature required to produce either of the two poisonous salts of mercury."

Messrs. Woodman and Tidy, in their work, on "Forensic Medicine and Toxicology," make the statement that the following medicines have sometimes produced salivation: Bromin, arsenic, prussic acid, nux vomica, cantharides, digitalis, conium, opium, and particularly iodid of potassium; and in the face of all this the mercury in amalgam fillings is made to bear so great a burden.

Professor A. Winter Blythe, in his work says, in speaking of amalgam, that the mercury is in *too powerful a state of combination* to be attacked by the fluids of the mouth.

That serious consequences will sometimes follow the insertion of any filling material placed in the cavity of a tooth, I will admit, but such results are not due to the *nature* of the *filling material*, but to the *condition* of the tooth at the time of filling. The first of these are caused by the pressure of the fillings on an exposed pulp; the second is where a filing is inserted over a devitalized pulp; and the third is where the pulp has been removed and the canals not properly sterilized.

The results from this method of fillings are facial neuralgia, shock from thermal changes, pain on percussion, pericementitis, alveolar abscess and great cellulitis, elevated temperature, fetid breath, and excessive flow of saliva, with corresponding constitutional depression. When amalgam had been used in a filling of this kind, all these conditions were said to be caused by the mercury in the amalgam, but *anything* that will hermetically seal a cavity containing septic material, will, on true surgical principles, elevate the temperature, the same as an abscess without a drainage-tube or any cavity in any part of the body containing septic material with no outlet.

The question that is constantly asked of me is, "How great a loss of mercury is sustained by an old amalgam filling ?"

This is most thoroughly exemplified by a wonderful series of scientific experiments, in a masterly work on that subject, and as the author of that book is in the room he will explain it far better than I can.

In a letter recently received from Dr. Jarvie he says, "I have practiced dentistry for thirty-five years, and have yet to see the first case where injurious results have attended the *intelligent use* of amalgam."

Far greater than any series of scientific experiments can possibly be, is the fact that tons and tons of amalgams are used every year, and no injury has ever been proven to be caused by it.

In J. Foster Flagg's work on "Plastics and Plastic Filling," in looking for some scientific experiments, he says :

"I found reference made to the practical experiments of Messrs. John and Charles Tomes, in 1861 to 1872, which I had regarded as conclusive and accepted as such. I found the term 'oxidization' as almost invariably applied to the discoloration of amalgam fillings, corrected by the acceptance of the long before urged, and much more reasonable hypothesis of 'sulphuretting.' I found a long list of elaborate experimentation with filled teeth and amalgam pellets, weighed with marvelous accuracy, placed in little bottles containing saliva acidulated with nitric, acetic, citric, or hydrochloric acid, and kept in a water-bath inside another water-bath at a uniform temperature—blood heat—for a period of three months, in order to prove by analysis of the saliva whether or not amalgam fillings would be capable of producing mercurial ptyalism. I could not reasonably doubt the certificate of the Professor of Analytical and Applied Chemistry that the saliva contained no mercury in solution."

In speaking of the systemic effects, the author quotes from a discussion held in the Pennsylvania Association of Dental Surgeons, as follows:

"Dr. C. N. Pierce, one of the professors of the Dental College of Pennsylvania, who opened the discussion, said in regard to amalgam, ptyalism, that it was a thing of 'so rare occurrence that he believed the profession had never heard of but one practitioner who thought that that result was produced by amalgam.' "

Professor T. L. Buckingham said, "he had never seen a case of salivation and had doubts about its ever having produced ptyalism;" that mercurial effects were "influences produced through the general system, and he did not think amalgam fillings would produce these effects."

Dr. J. H. McQuillen said that "in an experience of fourteen years he could not recall a single instance of necrosis of the jaws, ptyalism, etc., of which others assert they have seen so many;" and that while he recognized the fact of idiosyncrasies in which the smallest quantity of certain medicinal agencies is followed by untoward results, and would not, therefore, offer his negative testimony as positive proof, yet "his own experience had made him look upon those who assert that they have seen so many cases with considerable doubt as to the value of their judgment or opinions as reliable diagnosticians."

Dr. C. P. Fitch said, "in regard to its toxical or injurious effects upon the system, he was inclined to question, if not wholly doubt, any such influence, and concurred in the views advanced by Dr. McRuillen, that he had yet to see the first case of alveolar abscess, ptyalism, etc., due to the presence of mercury in the amalgam."

Dr. J. M. McGrath testified for himself and for his father, who had had an amalgam experience of ten or fifteen years, that as yet "they had never seen any bad effects resulting, such as had been ascribed to its use by many practitioners."

Dr. Flagg says: "Thus it was that I was fortified by the combined testimony of gentlemen whom I could esteem as conscientious observers, and for whom I had much regard, both socially and professionally. It now remained for me to add the testimony of almost thirty years more of increasingly acute scrutiny (this book was written ten years ago), with the assertion that during all of my amalgam experience *I have never seen one case* of mercurial ptyalism, mercurial periostitis, mercurial necrosis, or of the *slightest symptom which could reasonably be ascribed to mercurial action.* I have treated them experimentally with chlorate of potassium to demonstrate its utter impotency, and have then *cured every case* without the use of any anti-mercurials and have left the teeth refilled with amalgam. If anything more convincing than this is required, I have it not to offer."

I have before me a very elaborate series of experiments on "The Physical Properties and Physical Actions of Dental Amalgam," by E. A. Bogue, M. D., D. D. S., of New York, now known as Manhattan. If Dr. Bogue is present we should be very glad to hear from him.

In the first place, the dentine of the tooth has a circulation like a vegetable, and only that; the cementum of the tooth has a circulation like bone; the enamel, I need scarcely say, is skin to crystal. It is plainly stated in "Wood's Therapeutics" that minute doses of mercury increase the weight and increase the number of red blood corpuscles. So far, therefore, from being detrimental, it would seem that a thirty-eight years' wearing of amalgam fillings ought to have helped him. Perhaps it is that that enabled him to work eighteen hours a day! I do not see that such a supposition on my part would not be quite as susceptible of proof as the assumptions on Dr. Tuthill's part that these neuroses of which he speaks arose from amalgam fillings.

Then as to the power of mercury from amalgam to penetrate into the system. In the first place, the amalgam filling should be a chemical combination. It should not be a mere loose mechanical mixture. If it be a chemical combination, it is impossible to separate the mercury from the mass short of a heat very nearly approaching redness, excepting it be a dry heat. I did go through a series of experiments, which I have looked over tonight for the first time in twenty years, and I am ashamed of their elementary character, but at that time—in 1873—we knew very little of amalgam fillings. It so happened that two gentlemen then connected with Harvard University, and a third connected with the University of New Orleans had written certain papers which were read and discussed. In the course of my experimentation I kept a whole handful of amalgam fillings at 100° F. for three months, in saliva, for the purpose of making not only the test as to whether it were possible for mercury to be injurious systemically, but also for the purpose of finding out what the effects of certain chemicals were upon the teeth in connection with amalgam fillings. Perhaps, if I might be allowed, I will tell the reason that caused me to commence these experiments. A gentleman of great prominence in the now Greater New York placed his son in my hands for treatment and for a curious reason, as I supposed, declined to pay my bill. He sent him to some one else to have the amalgam fillings which I had put in taken out. This is history. It so happened I had not put any in. He then sent a letter from Dr. Metcalf, Professor of Theory and Practice at the College of Physicians and Surgeons, New

York, to me, with the statement that the probability was that his son was suffering from mercurialism from the presence of amalgam fillings. That boy had but one amalgam filling in his mouth, which had been there a number of years—a tiny thing, about as large as a pin's head. I replied to Dr. Metcalf that the boy was not suffering from mercurialization, but from chronic dyspepsia due to his inability to masticate properly—rather a saucy thing to say to a Professor of Theory and Practice. Dr. Metcalf took it kindly, however, and in a letter inquired of me whether it would not be possible for the emanations from amalgam fillings to produce systemic results. Thinking the question easy to answer I laid it by to the next day, and the next week, and in fact it was unanswered for twelve months, all of which time it took me, besides four or five hundred dollars in cash, before I was able to satisfy myself with an answer to that question. Then came the analysis made by Professor Chandler, cf Columbia College, in which, as has been stated, he certifies that there was no mercury to be found in any of the bottles of saliva, which for three months had been kept at 100° F., and contained, as I said before, a handful of amalgam such as dentists use. That report was made just after a little extract from the "Chicago Medical Journal" for July, 1873, had been put in my hands, in which was an article by Dr. Paine, in which he speaks of "the poisoning of thousands of people all over the world from corrosive sublimate generated in the mouth from amalgam plugs in the teeth; neither cholera, small-pox, or any malarious disease doing more injury in the world than this poison." That is broader and stronger than any statement that has been made this evening, and it was in answer to that and to Dr. Metcalf's very kindly question that these experiments were undertaken. I would only add that it is quite possible to eliminate mercury from amalgam fillings when they are dry, but when they are under saliva, or under water, it has not been found so in three-months' time.

I have been in practice for a number of years and have been looking for these cases of salivation. I saw last October a case that I thought was a typical one, but every filling in his mouth was gold. I saw another case only last month, and that man did not have a single filling in his mouth; so I hardly think that can be charged to the amalgam. The statement was made that amalgam fillings when removed are found to be bright on the side which comes in contact with the walls of the cavity. I think you will find they are as perfectly oxidized there, or "sulphuretted," as they are on the outside.

Mr. President, either mercury produces, when absorbed from the teeth, an entirely different clinical picture than when absorbed from the other parts of the system, or there is a discrepancy in Dr. Tuthill's cases. As he read them off they reminded me more of miscellaneous cases of neurasthenia and hysteria than anything else, and the rapidity with which they changed their character from time to time; the variability of the symptoms and the extreme rapidity with which they got well, even after existing for years. A mercurial poison which has existed for years almost invariably produces a continuous tremor, due to the deposit in the nervous system and accompanying sclerosis. Had these cases lasted for so many years there would have been the production of some organic change there and the candition would not have cleared up so quickly. I think the last speaker is quite correct regarding the element of mind-cure. I think that was the potent element which produced the cure in those cases.

We all admit that there is a circulation in the teeth which may be the means of carrying the poison through the system, and this kept up for weeks, months, years and decades, the system will feel the effect of this poison. I tell you, gentlemen, no one knows the direful consequences if he has not been there himself. Of course I am more interested in this subject, for it came near costing me my life.

I have often thought how the fathers in dentistry wrought well when they fought to the bitter end this subject of amalgam fillings; how they wrestled on the one side to prove it harmless, and on the other to expose its treachery and condemn its use. The struggle lasted from 1841 to 1847, when in the New York Dental Society all who had not pledged themselves not to use amalgam were obliged to resign or were expelled. Those who had been the best of friends became bitter enemies. I hope that the same conscientious feeling which prevailed then will prevail again.

I would like to say a word on that point. I think at present there is hardly a dentist in practice in the entire world but habitually uses amalgam; they have all overcome their prejudice to it. There is no more danger of that fight being renewed than of any other absurd thing.

I do not claim for this particular effect of mercury that you get salivation. There is no ptyalism present; I never saw it in one case, but, as I said in my paper, there is a peculiar action upon the nervous system. The theory that recovery was due to mind-cure is too absurd for consideration in connection with the cases I have cited. I have seen people who would get well on that theory, but I am sure that does not hold in these cases.—*Items of Interest.*

